# Different muscle strategy during head/knee level of functional reaching-transporting task to decrease falling probability in postmenopausal women with osteoporosis

**DOI:** 10.1186/s40945-023-00165-6

**Published:** 2023-05-09

**Authors:** Marzie Hatami, Giti Torkaman, Mohammad Najafi Ashtiani, Sanaz Mohebi

**Affiliations:** Physical Therapy Department, Faculty of Medical Sciences, Tarbiat Modares University, Ale-Ahmad Ave, P. O. Box: 1411713116, Tehran, Iran

**Keywords:** Osteoporosis‌‌, Postmenopausal Women, Reaching‌, Transporting, Electromyography, Muscle Activity

## Abstract

**Background:**

The reaching-transporting task as an essential daily activity impacts balance control and falling in older women. This study investigated the different muscle strategies during the head/knee level of the functional reaching-transporting task in postmenopausal women with osteoporosis.

**Methods:**

24 postmenopausal volunteers were classified into two groups based on the lumbar T-score: osteoporosis (≤ -2.5, *n* = 12) and non-osteoporosis (> –1, *n* = 12). Using a custom-designed device, participants randomly performed 12 reaching-transporting tasks at the head and knee levels. Electromyography signals were collected while reaching and transporting phases with a wireless system. The peak of the root means square (PRMS) and time to PRMS (TPRMS) were measured. In addition, the isometric muscle strength and the fear of falling were assessed.

**Results:**

The isometric muscle strength in the osteoporotic group was significantly lower than in the non-osteoporotic group (*P* < 0.05), except for vastus lateralis (VL). The PRMS of VL, (*P* = 0.010) during the reaching phase and VL (*P* = 0.002) and gastrocnemius lateralis (GL) (*P* < 0.001) during transporting phase was greater than the non-osteoporotic group. The PRMS value of the muscles was greater for reaching-transporting at the knee level than the head level; this increase was significant just for VL and biceps femoris during the transporting phase (*P* = 0.036 and *P* = 0.004, respectively).

**Conclusion:**

Osteoporotic women have more muscle activities during the reaching-transporting task, especially at the knee level, compared to the head level. Their muscle weakness may lead to insufficient stability during the task and cause disturbance and falling, which requires further investigation.


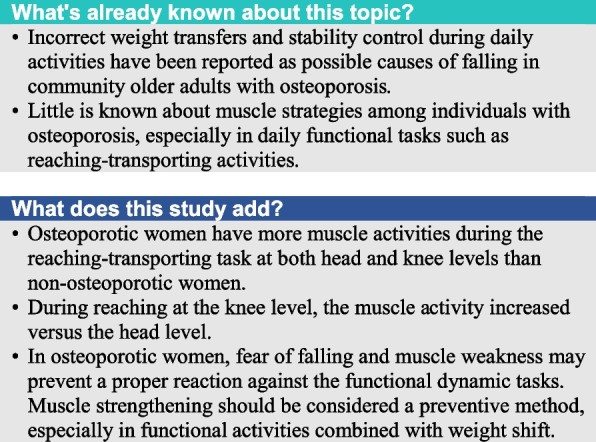


## Introduction

Osteoporosis is characterized by microarchitectural deterioration of bone tissues and bone mass loss, leading to bone fragility and susceptibility to fracture [1]. Fractures are more common in women than men, related to lower bone mass and less competing mortality, primarily in postmenopausal women, due to the osteoporosis associated with estrogen deficiency [2]. It was reported that, for women over 50 years, the lifetime risk of a fracture due to osteoporosis is 50% [3]. Fractures, as serious, costly events, result in disability and mortality; therefore, the study of fracture-related parameters to screen women at risk for fractures has been considered for a long time [4].

Falling is reported as the leading cause of eighty percent of all non-spine fractures and over ninety percent of hip fractures in older adults [5]. In this regard, postural stability has been proposed as an essential factor for functional independence in older adults, and impaired postural control is a major risk factor for falls [6]. Postural control is different in individuals with osteoporosis than in general older individuals. Individuals with osteoporosis are more likely to present higher sway velocities and a higher number of falls [7]. Furthermore, the fear of falling is common in women with osteoporosis-related to the knowledge of being at risk for falls and fractures, which may lead to restricted activity levels and an increased risk of falls [8, 9]. A study of community-dwelling older adults found that a substantial proportion of falls occurred during tasks such as carrying an object, reaching, or leaning [10]. Such actions as lean and reach, bending, stooping, and high reach tasks reportedly account for a substantial proportion of activities of daily living (ADL) in community-dwelling older adults [11]. These activities require shifting the center of gravity (CoG) within the base of support (BoS). Once the CoG moves outside the BoS, the stability limits for the currently executed balance strategy are exceeded. If the appropriate motor strategy is not implemented, the individual may stumble or fall in an attempt to regain balance [12]. Also, incorrect weight transfer and stability control during these voluntary movements have been reported as a possible cause of falling [13] and fractures in older adults. Osteoporotic people with bone fragility are probably at greater risk of fractures due to falling contributed to such incorrect strategies, especially while performing high-risk tasks based on evidence, such as reach-lean-bending and stooping tasks [14].

As a clinical estimator of the stability margin, it was demonstrated that the functional reach (FR) test could predict falling associated with impaired dynamic balance [15]. However, despite extensive use of the FR test, inconsistent results restricted its efficacy as a clinical tool to assess dynamic balance and fall risk [16, 17]. For detecting individuals at risk of falling, the accuracy of the FR test may be increased in more functional situations, such as accompanied by a subsequent grasp and lift or transport of an object [18]. In addition to the above, the greater muscle coactivation at the ankle joint (increase in ankle rigidity), associated with the aging preceding the reaching task, has a detrimental effect on dynamic postural control due to the restriction of efficient postural adjustment to the perturbations and consequently increases the risk of falling [19]. Increasing the simultaneity of antagonistic activity, which causes joint stiffness, is a protective strategy to compensate the poor postural control. This strategy can be due to muscle weakness, reduction of fast muscle fibers, and other sensory-motor disturbances in older adults [19].

Older adults use unique balance strategies related to postural instability during ADL, such as more reliance on hip strategy and increased muscle coactivation that affect static and dynamic postural control ability [18, 19]. The reaching-transporting task as an essential component of daily activities can challenge the balance control of these people due to the production of joint torque and changes in posture. Recording the activity of leg muscles, which may have a crucial role in maintaining the upright posture and consequently controlling the center of pressure (CoP) within BoS [20], may determine the muscle contracting strategies during FR and transporting tasks in people with osteoporosis.

To our knowledge, little is known about muscle strategies and postural control among seniors with osteoporosis, especially in ADL tasks such as reaching-transporting activities. To our knowledge, the differences in the electromyography (EMG) of leg muscles have not been investigated in postmenopausal osteoporotic women while reaching and transporting objects at different heights. Thus, the present study investigated the EMG activity of leg muscles during the reaching-transporting task at head and knee levels in osteoporotic and non-osteoporotic postmenopausal women. We proposed bone mineral density (BMD) and level of reaching interact with the EMG activity of two paired agonist–antagonist groups composed of vastus lateralis (VL)- biceps femoris (BF) and tibialis anterior (TA)- gastrocnemius lateralis (GL), during the execution of the FR and transport task.

## Material and methods

### Participants

This experimental research was an analytical cross-sectional study. It was a single-blind study in which the assessor was blinded to the grouping. It was conducted with 24 postmenopausal women volunteers at the motion analysis laboratory of the physical therapy department Tarbiat Modares University. The Medical Ethics Committee of Tarbiat Modares University approved the study (IR.MODARES.REC.1398.068). Forty postmenopausal women responded to the advertising notices posted via social networks between April 2019 and July 2019. All 40 volunteer women were screened, and based on the inclusion and exclusion criteria, 24 people were included in the study. The cochranʼ formula estimated the sample size through mean and standard deviation values of the normalized EMG activity of the TA in the FR test, in the study of Nagai et al., with a 95% confidence interval and 80% power [21]. The inclusion criteria included females 50–70 years of age, menopausal for at least one year before the study (losing their monthly menstrual cycle for at least one year, which a gynecologist approved), with no record of regular physical exercise for at least one year. Exclusion criteria were: secondary osteoporosis, a history of osteoporotic fracture, a history of falling during the year before the experiment, thoracic kyphosis as a Cobb angle more than 50º, genu valgus with the inter-malleolar distance of more than 8 cm, or genu varus with inter-condylar distance more than 6 cm, the presence of neurogenic or myopathy disorders, diabetes, thyroid disease, rheumatoid diseases, any malignant neoplasia. After being informed about the study, all participants gave their written consent to the experimental procedures. The participants were classified into two groups based on the lumbar BMD status: osteoporosis (T-score ≤ -2.5, *n* = 12) and non-osteoporosis (T-score > –1, *n* = 12). Dual-energy x-ray absorptiometry was performed to measure BMD scores for all participants in the same radiography center. An expert blinded physiotherapist performed all assessments from 9 a.m. to 1 p.m. In addition, the maximum voluntary isometric contraction (MVIC) was recorded. The fall efficacy scale (FES) was administered through the Persian version of the fall efficacy scale questionnaire and was used as a self-report. The Persian-translated version of the FES has acceptable validity and reliability for the Iranian older adult population [22].

### Experimental setup and protocol

#### Maximum voluntary isometric contraction

For each participant, MVIC of the knee extension and flexion and ankle dorsi and plantar flexion was measured on the dominant side. The measurement was done by a digital hand-held dynamometer (Hand-held Dynamometer; Lafayette Instrument Co., Lafayette, IN, USA), recording the muscular strength from 0.2 to 199.9 kg. The Make method recorded the measurements in kilograms (kg) because of its repeatability of 0.909 [23]. The MVIC holding was 5 s, and each muscular group was tested thrice with 30 s rest between them. A blinded expert physiotherapist did all measurements, and the average value was used for the subsequent analyses. The procedure, which included test positions, stabilized regions, and dynamometer placements, was based on Bohannon [24]. In addition, during the MVIC test, the EMG signal of the target muscle was recorded to normalize the muscle activity during the reaching-transporting task.

#### EMG recording and processing

EMG signals were collected with a 16-channel wireless EMG system (Aktos, Myon Inc., Switzerland). The skin of the dominant leg was shaved and then cleaned with alcohol. Wireless surface electrodes were placed on the VL, BF, TA, and GL according to the SENIAM [25]. The sampling frequency was 1200 Hz with a 20 – 450 Hz bandpass. Data gathering was done during MVIC and the reaching-transporting task. The raw signal was filtered with a 2^nd^ order Butterworth filter, rectified, and normalized to the MVIC. We calculated the root mean square value (RMS) in 100-ms windows. During the reaching and transporting phases, the peak RMS (PRMS) and time to the peak RMS (TPRMS) were extracted using a custom script developed in MATLAB™ R2018B (MathWorks Inc, Natick, MA).

#### Experimental reaching -transporting task

**Fig. 1:**
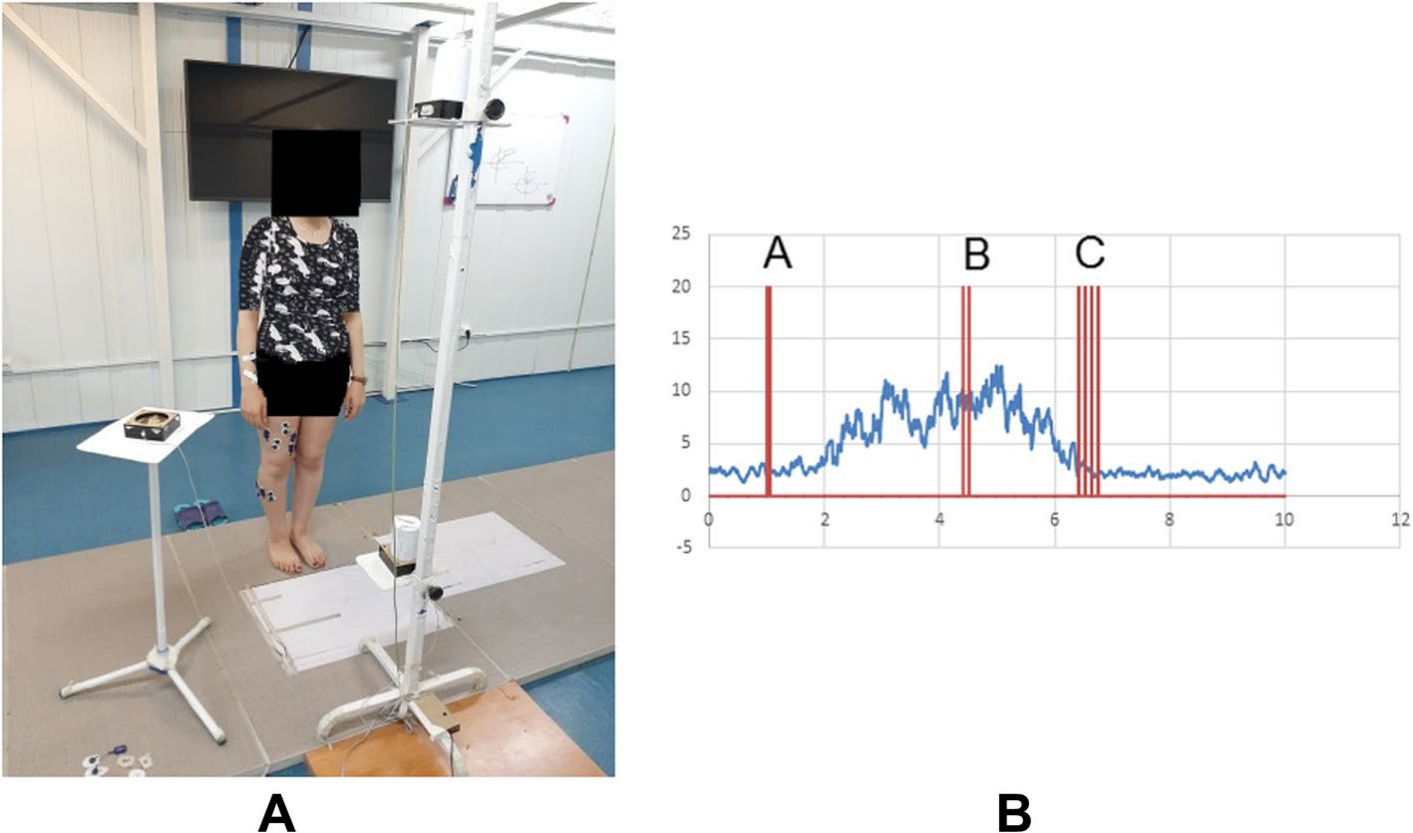
The setup of the reaching-transporting task (**A**), **B**; A sample of the EMG signal of the tibialis anterior muscle when reaching-transporting task performed at head level. Spike A is the time of start comment (LED on); **B** is the time of removing the object (can) from the head level; **C** is the time of putting the can on the lateral platform

A custom-made object stand was designed with two adjustable platforms subjectively at the two-level of the head and knee (Fig. 1A). The volunteers stood 110% of their upper limb length away from the stand. They performed a series of object transport tasks barefoot with a fixed support base. The distance between heels was set at 10% of the height. A lateral platform with a height of 90 cm was placed on the dominant side of the participants; at two o’clock or ten o’clock positions for the right and left-handed participants, respectively. A can of 8 cm diameter and 400 gr weight was used for transporting tasks. Each participant was instructed to remove the can from the platforms, determined randomly by a visual command through a light-emitting diode (LED) installed in the front of each head/knee platform. Then, place it on the lateral platform. The participants were asked to perform the task immediately after turning the light on at the fastest safe speed that would not disturb their balance. Each participant randomly performed 12 reaching-transporting tasks, six times at the head and six at the knee levels. At the surface of each platform, a micro-switch was installed to record removing or putting the object. These signals, as the spike, were triggered on the EMG signals (Fig. 1B); A-spike, comment to ready, B-spike taking the object at the head (two pulses included) or knee level (three pulses included), and C-spike, putting the object on the lateral platform. The time duration between A and B-spikes was considered the reaching phase, and between B and C-spikes was supposed to be the transporting phase.

### Statistical analysis

The Shapiro–Wilk test was used to test the normal distribution of data. So, the independent-sample *t*-test was used to compare the anthropometric variables, FES score, and MVIC between the two groups. The general linear univariate model (GLUM) was used to determine the effect of grouping (BMD status, osteoporosis / non-osteoporosis) and level of reaching (head/knee level) on the leg muscle activity. Statistical significance was set at *P* ≤ 0.050. IBM SPSS Statistics version 16(IBM, Armonk, NY, USA) was used for statistical analysis.

## Results

Table 1 shows no significant differences in age and BMI (*P* > 0.05) between the two groups. Hip and Spine T-scores were significantly lower in the osteoporotic group compared to the non-osteoporotic (normal BMD) group (*P* < 0.05). The fall efficacy score was significantly higher in the osteoporotic group than in the non-osteoporotic group (*P* < 0.001). The MVIC in the osteoporotic group was significantly lower than that in the non-osteoporotic group (*P* < 0.05), except for vastus lateralis, which was not significant (*P* = 0.126).

**Table 1:** Anthropometric and demographic characteristics and MVIC in two groups; Mean (SD)

Variables	Osteoporotic (*n* = 12)	Non-osteoporotic (*n* = 12)	*P*-Value
Age (years)	60.33 (7.03)	58 (5.39)	0.372
BMI (kg/m2)	25.04 (1.53)	26.32 (2.21)	0.062
T-score			
Hip T-score	**-1.89 (0.53)**	**1.37 (0.58)**	0.034^*^
Spine T-score	**-2.99 (0.44)**	**1.53 (0.39)**	0.042^*^
FES	**49 (12.01)**	**24.58 (7.91)**	0.001^*^
Maximum Voluntary Isometric Contraction (Kg)			
Tibialis anterior	**4.44 (0.57)**	**4.95 (0.62)**	0.048^*^
Gastrocnemius lateralis	**4.36 (1.33)**	**5.35 (0.79)**	0.039^*^
Biceps femoris	**4.93 (1.22)**	**7.39 (0.51)**	< 0.001^*^
Vastus Lateralis	8.9 (0.77)	8.1 (1.62)	0.126

^*^ Significant difference between two groups (*P* ≤ 0.05). *BMI* Body Mass Index, *FES* Fall Efficacy Scale

**Fig. 2:**
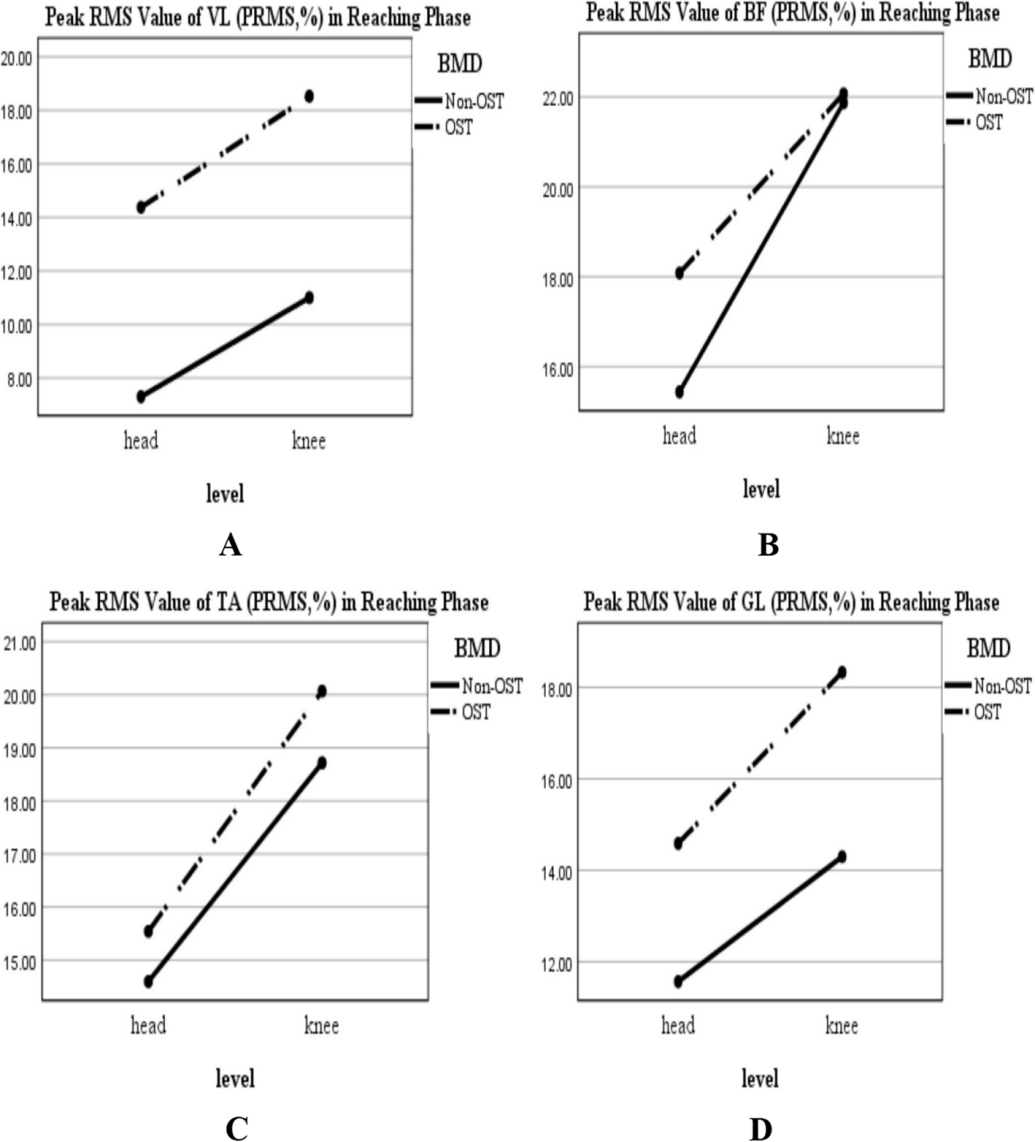
BMD and level of reaching interaction plots for the general linear univariate model. **A** The reaching PRMS of VL. **B** The reaching PRMS of BF. **C** The reaching PRMS of TA. **D** The reaching PRMS of GL. Non-OST: Non-osteoporotic, OST: Osteoporotic, Peak RMS: Peak Root Mean Square value in Reaching phase (PRMS in percent (normalized value)), VL: Vastus Lateralis; BF: Biceps Femoris; TA: Tibialis Anterior; GL: Gastrocnemius Lateralis

**Fig. 3:**
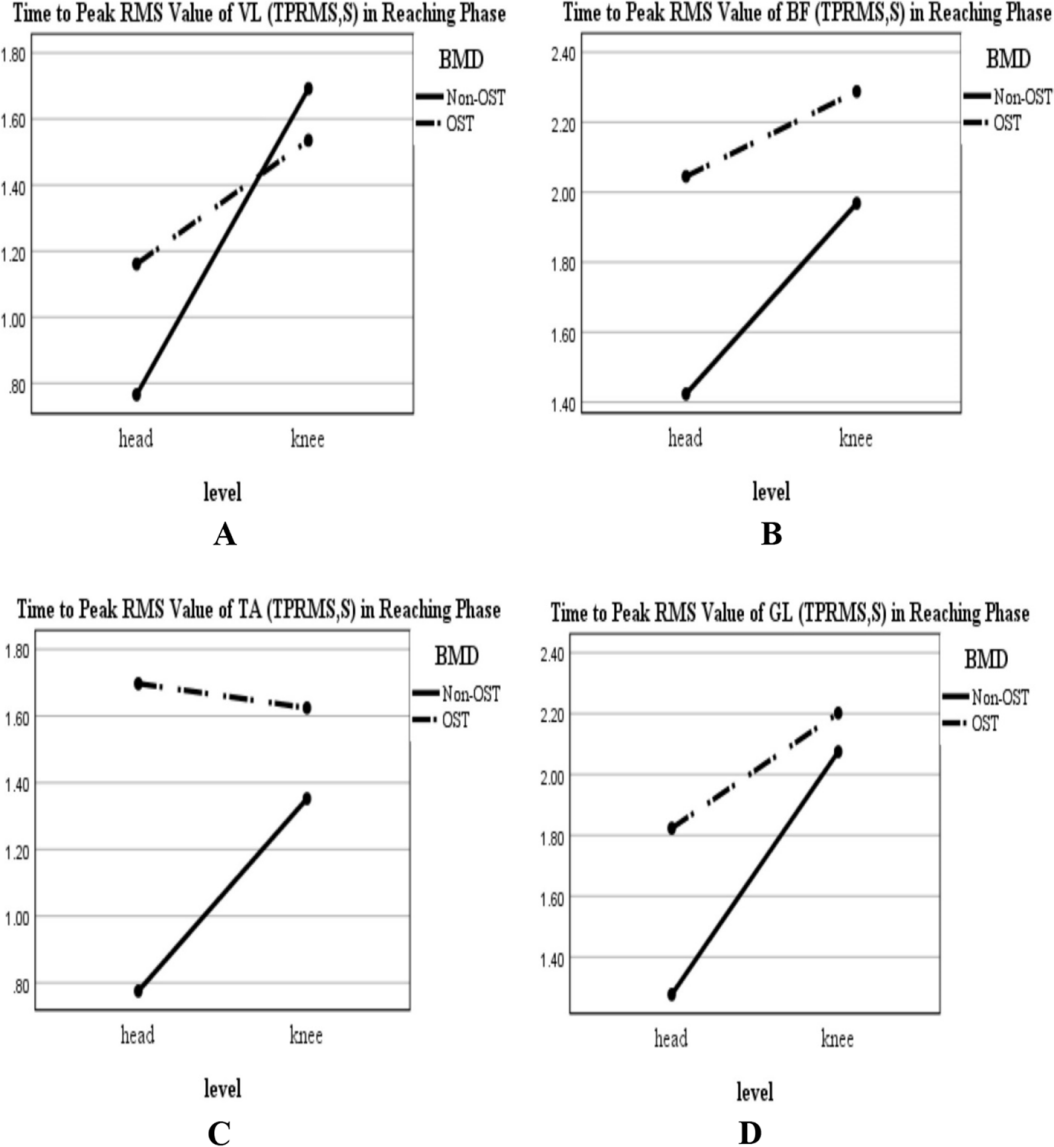
BMD and level of reaching interaction plots for the general linear univariate model. **A** The reaching TPRMS of VL. **B** The reaching TPRMS of BF. **C** The reaching TPRMS of TA. **D** The reaching TPRMS of GL. Non-OST: Non-osteoporotic, OST: Osteoporotic; TPRMS: Time to Peak RMS value (s); VL: Vastus Lateralis; BF: Biceps Femoris; TA: Tibialis Anterior; GL: Gastrocnemius Lateralis

**Fig. 4:**
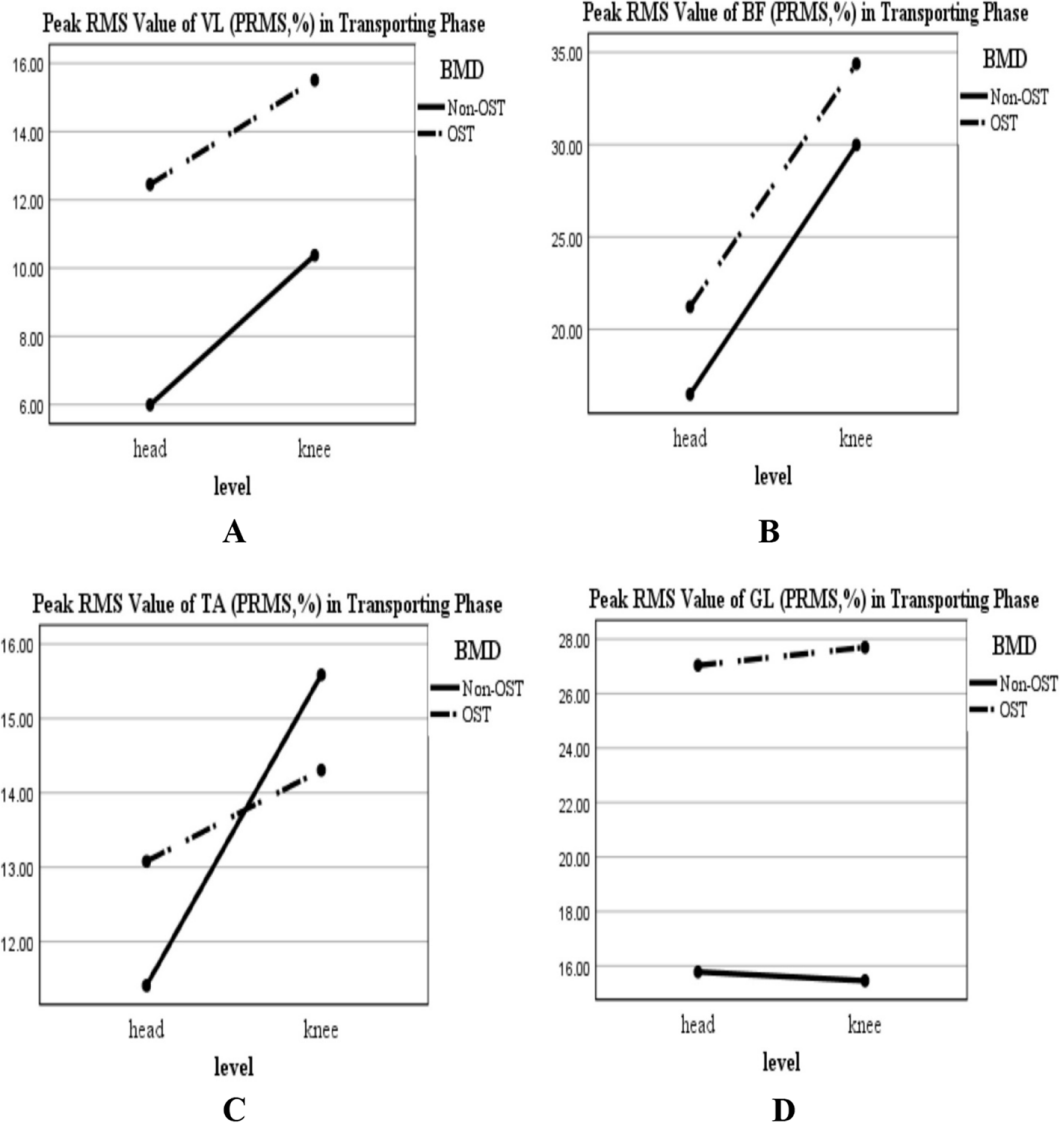
BMD and level of reaching interaction plots for the general linear univariate model (during transporting phase). **A** PRMS of VL. **B** PRMS of BF. **C** PRMS of TA. **D** PRMS of GL. Non-OST: Non-osteoporotic, OST: Osteoporotic, Peak RMS: Peak Root Mean Square value in Transporting phase (PRMS in percent (normalized value)), VL: Vastus Lateralis; BF: Biceps Femoris; TA: Tibialis Anterior; GL: Gastrocnemius Lateralis

**Fig. 5:**
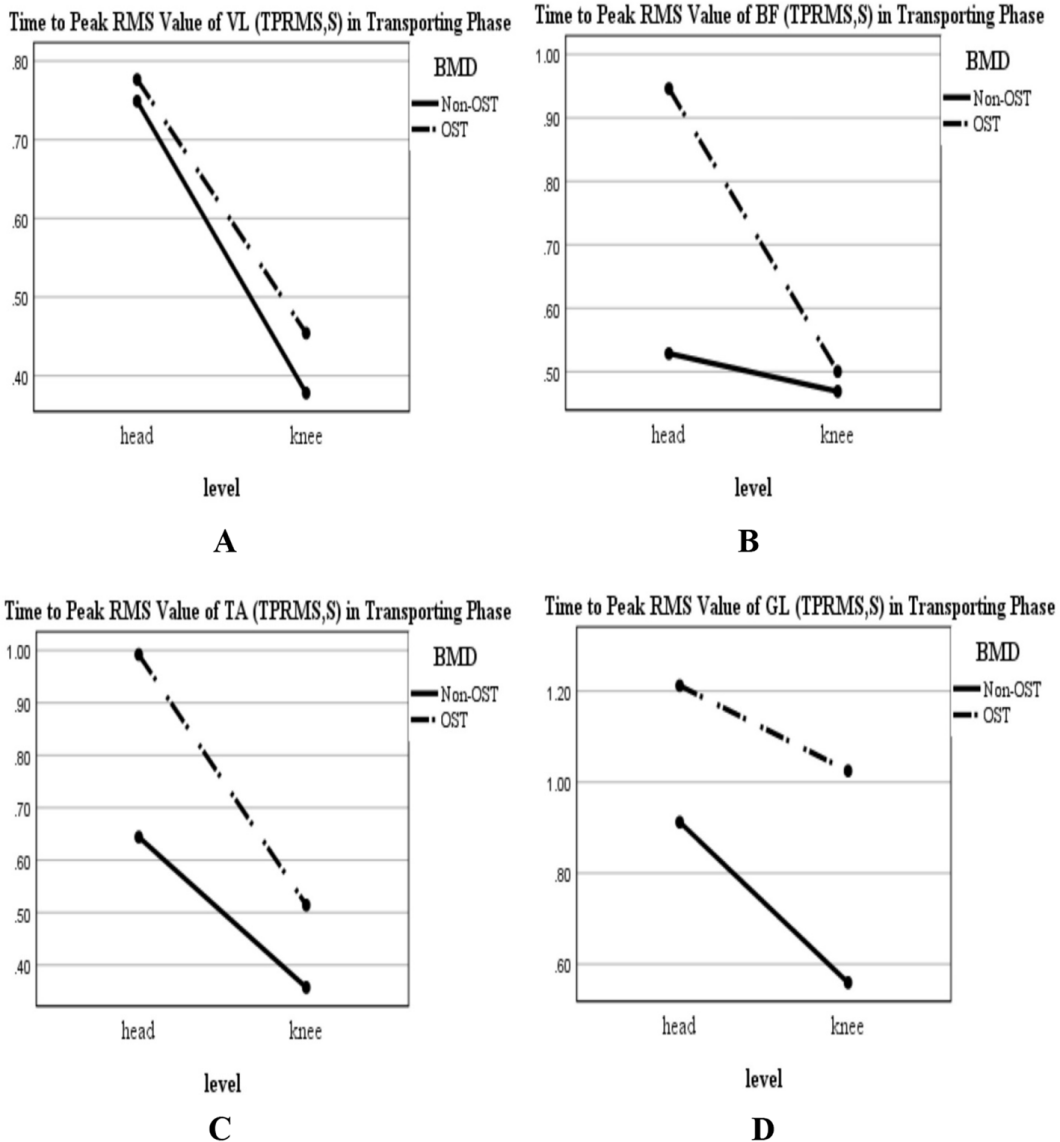
BMD and level of reaching interaction plots for the general linear univariate model (during transporting phase). **A** TPRMS of VL. **B** TPRMS of BF. **C** TPRMS of TA. **D** TPRMS of GL. Non-OST: Non-osteoporotic, OST: Osteoporotic; TPRMS: Time to Peak RMS value (s); VL: Vastus Lateralis; BF: Biceps Femoris; TA: Tibialis Anterior; GL: Gastrocnemius Lateralis

According to Figs. 2 and 4, regardless of BMD condition, the PRMS value of the muscles was greater for the reaching-transporting at the knee level compared to the head level; however, this increase was significant just for VL and BF during transporting phase (Table 2; F-value: 4.69, *P* = 0.036; Fig. 4A; F-value: 9.08, *P* = 0.004; Fig. 4B, respectively). In this regard, TPRMS values increased significantly in VL and GL during the reaching phase (Table2; F-value: 12.11, *P* = 0.001, Fig. 3A; F-value:8.22, *P* = 0.006, Fig. 3D, respectively), and decreased significantly for VL, BF, TA, and GL during transporting phase (Table2; F-value: 10.87, *P* = 0.002, Fig. 5A; F-value:6.57, *P* = 0.014, Fig. 5B; F-value: 16.39, *P* < 0.001, Fig. 5C; F-value: 10.77, *P* = 0.002, Fig. 5D, respectively).

**Table 2:** Results of the general univariate linear model for the main effect of BMD and Level of task on the PRMS and TPRMS

*PRMS (normalized to MVIC in percent)*	**BMD condition**				**Level of reaching**			BMD* level of reaching			
	F Statistic			*P*-Value	F Statistic	*P*-Value		F Statistic	*P*-Value		
	**Reaching Phase**										
**Vastus Lateralis**	**7.32**			0.010^*^	2.11	0.153		0.007		0.934	
**Biceps femoris**	0.178			0.676	2.37	0.130		0.131		0.719	
**Tibialis Anterior**	0.89			0.766	1.26	0.267		0.003		0.958	
**Gastrocnemius Lateralis**	2.60			0.114	2.19	0.146		0.530		0.818	
*TPRMS(s)*											
**Vastus Lateralis**	0.41		0.525		**12.11**		0.001^*^	2.18		0.147	
**Biceps femoris**	**5.33**		0.026^*^		3.73		0.060	0.550		0.462	
**Tibialis Anterior**	**6.55**		0.014^*^		1.17		0.285	1.94		0.170	
**Gastrocnemius Lateralis**	2.69		0.108		**8.22**		0.006^*^	1.04		0.312	
*PRMS (normalized to MVIC in percent)*	**Transporting Phase**										
**Vastus Lateralis**	**11.39**	0.002^*^			**4.69**		0.036^*^	0.150			0.701
**Biceps femoris**	1.06	0.307			**9.08**		0.004^*^	0.002			0.969
**Tibialis Anterior**	0.005	0.203			1.014		0.319	0.302			0.585
**Gastrocnemius Lateralis**	**14.74**	< 0.001^*^			0.003		0.956	0.026			0.873
*TPRMS(s)*											
**Vastus Lateralis**	0.24	0.624			**10.87**		0.002^*^	0.053			0.818
**Biceps femoris**	**5.18**	0.028^*^			**6.57**		0.014^*^	3.83			0.057
**Tibialis Anterior**	**7.15**	0.010^*^			**16.39**		< 0.001^*^	1.024			0.317
**Gastrocnemius Lateralis**	**21.62**	< 0.001^*^			**10.77**		0.002^*^	1.01			0.320

^*^Significant effect, *BMD* Bone mineral density, *PRMS* the peak root mean square, *TPRMS* time to the peak root mean square

Also, regardless of the level of the reaching-transporting task, the PRMS value of the muscles was greater for the osteoporotic group compared to the non-osteoporotic group (Figs. 2 and 4); this increase was significant for VL during the reaching phase (Table2; F-value: 7.32, *P* = 0.010; Fig. 2A) and for VL and GL during transporting phase (Table2; F-value: 11.39, *P* = 0.002; Fig. 4A; F-value: 14.74, *P* < 0.001; Fig. 4D, respectively). In this regard, the TPRMS values generally were longer in the osteoporotic group than in the non-osteoporotic group (Figs. 3 and 5). This increase was significant for BF and TA during the reaching phase (Table2; F-value: 5.33, *P* = 0.026, Fig. 3B; F-value:6.55, *P* = 0.014, Fig. 3C, respectively) and for BF, TA, and GL during transporting phase (Table2; F-value: 5.18, *P* = 0.028, Fig. 5B; F-value:7.15, *P* = 0.010, Fig. 5C; F-value:21.62, *P* < 0.001, Fig. 5D, respectively). BMD* Level of reaching-transporting showed no significant interaction (Table 2; *P* > 0.05).

## Discussion

The primary purpose of this study was to investigate the interaction of BMD status and the level of reaching-transporting tasks (head/knee) on leg muscle activities. Also, we assessed FES and MVIC based on their importance in postural control. It can be helpful to know this relationship for prescribing rehabilitation exercises and the ergonomic design of the home environment for osteoporotic women at risk of postural instability, falling, and fractures.

In the reaching phase, our results showed that the PRMS and TPRMS were generally greater in the leg muscles of the osteoporotic individuals regardless of the reaching level. Furthermore, according to the GLUM analysis, the activity of the VL in the reaching phase was significantly associated with the BMD condition; the PRMS was higher in osteoporotic participants at both reaching levels.

In the reaching phase, when the volunteers shifted their center of mass (CoM) anteriorly, greater activation of posterior leg muscles, as agonist’s muscles, is needed to control the smooth forward movement of CoM. Therefore, the unexpected increased activity of VL might be due to a coactivation strategy in osteoporotic participants during the reaching phase. In this way, it was reported that older adults with postural control deterioration utilize muscle coactivation strategies to increase joint stiffness and enhance stability [21, 26]. Our results confirmed the significant decrease in leg muscle strength in osteoporotic women. In addition, the osteoporotic women reported higher fear of falling scores than non-osteoporotic women. According to Nagai et al., fear of falling is associated with increased muscle coactivation during walking [27]. Hence, conservative stiffening strategies, such as muscular coactivation, the higher internal focus of attention, further motor unit recruitment to provide required force by weak muscles, reduction of the range of motion and joint angular velocities, and slower and more purposeful movements probably related to the higher fear of fall in the older adults [28]. When the reaching task was performed at the knee level, regardless of BMD condition, the PRMS values (not significantly) and TPRMS values (significantly for VL and GL) increased versus the head level. During the reaching task at the knee level, the postural control system might trigger more protective strategies to reduce the postural instability and falling risk. As a result, reaching may be done cautiously and with more muscle activation (increasing the PRMS) to control stability which causes a delay in reaching the PRMS.

In transporting phase, regardless of the reaching level, the PRMS of the muscles (significant for VL and GL) and related TPRMS values (significant for BF, TA, and GL) were greater in the osteoporotic group. Thus, the VL and GL activity increase was significantly related to osteoporosis. In this phase, the essential kinematic component is the external rotation of the lower limb to transport and place the object on the lateral table, regardless of the reach level. Therefore, the increase of GL activity as a distal leg external rotator and VL as a proximal leg internal rotator might be related to a coactivation strategy to provide proximal stability during lower extremity motion and a reciprocal strategy in distal leg muscles to give the critical moment during transport of the object. According to Kanekar et al., in older adults, the coactivation pattern between the trunk and thigh muscles was observed as a more conservative strategy to maintain postural stability [29].

Therefore, the muscle weakness and increased recruitment of motor units related to the co-contraction strategy may explain the increase of PRMS and related TPRMS values recorded in the transporting phase, under the main effect of osteoporosis. Our results also indicated that in transporting phase at the knee level, regardless of BMD condition, the PRMS values (significantly for VL and BF) increased, and TPRMS values (significantly for VL, BF, TA, and GL) decreased than the head level. This increase in PRMS and decrease in TPRMS values might be related to recruiting larger and faster motor units to provide the higher hip and knee extensor moments required to transport the object from the knee level to the lateral platform. In general, while the object is transported from the knee level, increased PRMS values and decreased TPRMS values may be associated with preferential recruitment of larger and faster motor units with higher force, discharge rate, and peak shortening velocity [30, 31]. Increased leg muscle activity may preferentially recruit the larger and faster motor units, and conservative behaviors in a more unstable situation, such as the reaching-transporting at the knee level, may be associated with a reduced risk of falling.

Of course, it should be noted that the higher muscle activity in the osteoporotic participants, with weaker muscles, at both head and knee levels, may lead to excessive energy expenditure, resulting in fatigue and postural instability. On the other hand, excessive muscle coactivation increases postural rigidity and might restrict dynamic postural control, especially in daily activities with probable unpredictable perturbations [32, 33]. Muscle fatigue was not assessed in this study. Of course, the proposed few reach-transport tasks rarely lead to leg muscle fatigue. Muscle fatigue might happen in activities of daily living with more repetition and longer time and during performing tasks with excessive muscle activation.

In addition to muscle weakness, the greater fear of falling in osteoporotic women may be related to an increased risk of instability, falling, and fractures due to the different strategies this population utilizes while reaching and transporting an object. So, lower limb muscle strengthening and cognitive-behavioral interventions to reduce fear of falling and related activity restriction may reduce the risk of falls associated with the changes in the lower extremity muscle activity patterns observed in osteoporotic women [34].

Overall, the results showed that the muscles in the osteoporotic group are weaker, and they need higher muscle activity to reach and transport an object. Due to muscle weakness, this activity may not maintain stability during the task and may cause disturbance and falling. In addition, osteoporotic women have more muscle activity during reaching-transporting the object from the knee level than from the head level. It may be due to bending forward to pick up the object that needs higher effort to maintain stability. This situation can increase the possibility of falling because muscle weakness will prevent a proper reaction against this perturbation. Muscle strengthening, especially in dynamic functional activities combined with weight shift, can be considered a prevention method for osteoporotic women. Of course, performing the reaching-transporting task at high speed is probably more effective in showing the different control strategies between osteoporotic and non-osteoporotic women, which should be considered in future studies.

The main limitation of this study was the small sample size; it suggests performing with a larger sample size for external validity. Faller women were not included in the present study. In future studies, comparing muscle strategy during the reaching-transforming task in faller and non-faller osteoporotic women is recommended. Due to the importance of trunk muscles in controlling weight-shifting tasks, it is suggested to record the activity of the trunk muscles during the FR-transport task. The other limit was the lack of simultaneously recording the CoP sway, which is recommended to consider in future studies. It helps to obtain more details about the altered CoP strategies in osteoporotic women during the reaching-transporting task.

## Conclusion

It is concluded that the muscle’s PRMS values recorded during the reaching and transporting of objects at the head and knee levels were higher in osteoporotic compared to age-matched non-osteoporotic individuals. It may contribute to the coactivation strategy utilized by osteoporotic women due to the weakness of the muscles and the higher fear of falling. An increase in PRMS and decreased TPRMS values at the knee level task might be associated with recruiting the larger-faster motor units to provide higher extensor moments required to extend the lower extremity for transporting tasks. These results can help design the prevention rehabilitation program and ergonomic considerations for the arrangement of household items. Having information about the more stable situation of reaching-transporting tasks and cautious strategies through increasing the leg muscle activity may be helpful to better design household arrangements. For example, placing shelves and household items at an appropriate level might be a safe, ergonomic strategy to prevent falling in osteoporotic women, although it needs more investigation to approve. Standing weight transfer training at different levels and during functional activity is recommended for osteoporotic women to decrease the risk of falling.

## Data Availability

The datasets generated and analyzed during the current study are available from the corresponding author on reasonable request.

## Abbreviations

BF: Biceps Femoris
BMD: Bone mineral density
BMI: Body mass index
BoS: Base of Support
CoG: Center of Gravity
CoM: Center of Mass
CoP: Center of pressure
EMG: Electromyography
FR: Functional Reach
GL: Gastrocnemius Lateralis
GLUM: General Linear Univariate Model
MVIC: Maximum Voluntary Isometric Contraction
R.Rms: Peak Root Mean Square value in Reaching phase (PRMS in percent (normalized value))
TA: Tibialis Anterior
Tr.Rms: Peak Root Mean Square value in Transporting phase (PRMS in percent (normalized value))
VL: Vastus Lateralis
